# Hereditary Protein S Deficiency and Activated Protein C Resistance Manifesting With Recurrent Thrombosis and Stroke

**DOI:** 10.7759/cureus.34012

**Published:** 2023-01-20

**Authors:** Josef Finsterer

**Affiliations:** 1 Neurology, Neurology and Neurophysiology Center, Vienna, AUT

**Keywords:** protein s, factor v leiden, arterial occlusion, thrombosis, coagulopathy

## Abstract

The dual coagulation disorder hereditary protein S deficiency and activated protein C (APC) resistance, which clinically manifests with recurrent venous thrombosis and multifocal ischemic stroke, has only rarely been reported in the same patient. The patient is a 54-year-old male with a history of recurrent, asymptomatic ischemic stroke or transient ischemic attack (TIA) since age 14 and four episodes of deep vein thromboses (DVT), two complicated by pulmonary embolism, attributed to hereditary protein S deficiency and homozygous factor V Leiden mutation. In addition, the medical history was positive for obesity, previous chronic alcoholism, smoking, gynecomastia with left breast resection, arterial hypertension, hepatic steatosis, and cholecystolithiasis. Because of low compliance, long-term oral anticoagulation with phenprocoumon from the age of 38 was replaced by dabigatran (300 mg/d) after another stroke with bleeding at the age of 54. In summary, the simultaneous presence of two hereditary coagulation disorders can lead to multiple venous thromboses and recurrent ischemic stroke. An appealing therapeutic option in poorly compliant patients with these two hereditary clotting defects is the replacement of long-term anticoagulation with a vitamin K antagonist (VKA) by a direct oral anticoagulant.

## Introduction

Protein S deficiency is a rare coagulation disorder characterized by reduced activity of protein S, which is involved not only in coagulation but also in inflammation and apoptosis [1]. Protein S facilitates the action of activated protein C (APC) on activated factors 5 and 8 [1]. A deficiency of protein S leads to an inability to control clotting and excessive formation of blood clots in the venous and arterial vascular beds [1]. Protein S deficiency can be either hereditary or acquired due to liver disease, nephrotic syndrome, or vitamin K deficiency [1]. Thrombosis occurs with both homozygous and heterozygous mutations in the PROS1 gene [1]. Factor V Leiden thrombophilia (APC resistance) is an inherited coagulopathy due to variant c.1691G>A in F5 (factor V gene) [2]. The Leiden mutation leads to a weak anticoagulant response to APC [2]. Heterozygosity for the mutation has little impact on the risk of thrombosis, but homozygous carriers have a much greater risk of thrombosis [2]. Both hereditary coagulation disorders have only rarely been reported in the same patient [3-5]. Here, we report a patient with hereditary protein S deficiency and APC resistance, which manifested with multiple venous thromboses, intraventricular thrombi, and multiple ischemic strokes.

## Case presentation

The patient is a 54-year-old Caucasian male, 170 cm tall and weighing 170 kg (BMI: 58.8 kg/m^2^), with hereditary protein S deficiency and homozygous APC resistance, and a history of recurrent venous thromboses and recurrent ischemic stroke. At the age of 14, he suffered a transient ischemic attack (TIA), which presented with speech arrest and weakness of the left upper limb but resolved completely within 24 hours after onset without further examination regarding the cause or prescription of aspirin. At the age of 36, without taking medication regularly, he developed his first deep vein thrombosis (DVT) in the left lower extremity, which is why he was anticoagulated with phenprocoumon for six months. At the age of 38, he suffered a second DVT, again located in the left lower extremity, this time associated with a pulmonary embolism. Lifelong anticoagulation with phenprocoumon was started. At age 42, despite adequate anticoagulation, a third DVT occurred, this time in the right lower extremity, again complicated by pulmonary embolism. Treatment with phenprocoumon was maintained. At the age of 46, he suffered a fourth DVT, this time again in the left lower extremity without pulmonary embolism. His history was further positive for arterial hypertension, obesity since age 52, previous alcoholism (up to age 44), smoking, anemia, gynecomastia, hepatic steatosis, and cholecystolithiasis but negative for stroke. He was on a walker at home. His family history was positive for protein S deficiency in his mother’s sister. His regular home medication included phenprocoumon (alternatively 1 and ½ tablets every other day for three years without regular international normalized ratio (INR) monitoring).

**Figure 1:**
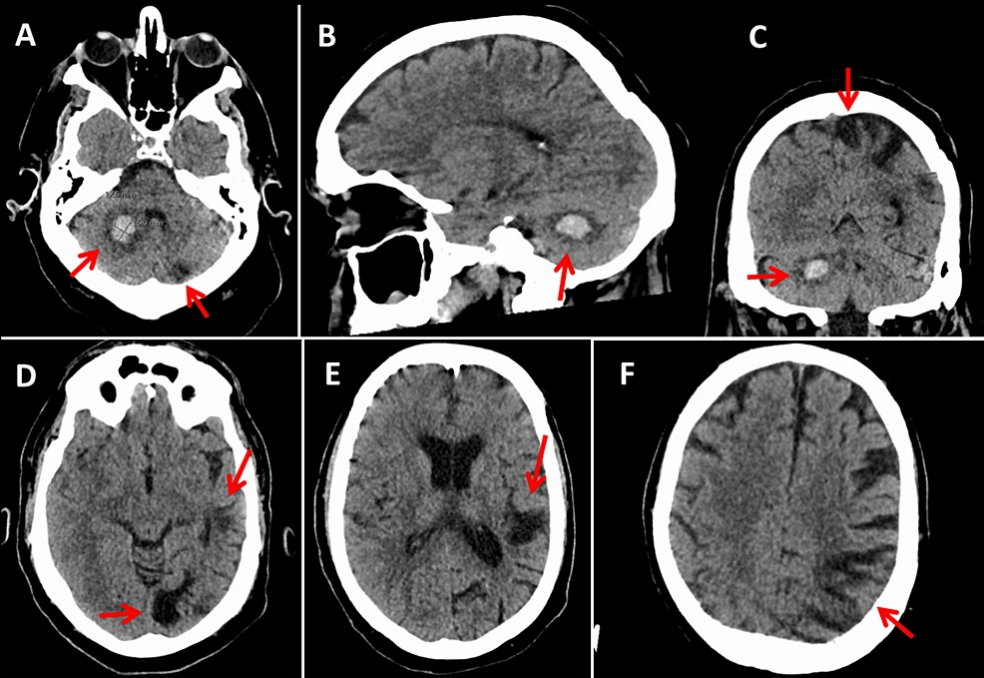
Cerebral CT showing an ischemic stroke with secondary bleeding in the right cerebellar hemisphere (A-C) (red arrows). In addition, old ischemic lesions can be seen in the area of the left posterior cerebral artery (D) and the left middle cerebral artery (D and F) (red arrows). CT: computed tomography

**Figure 2:**
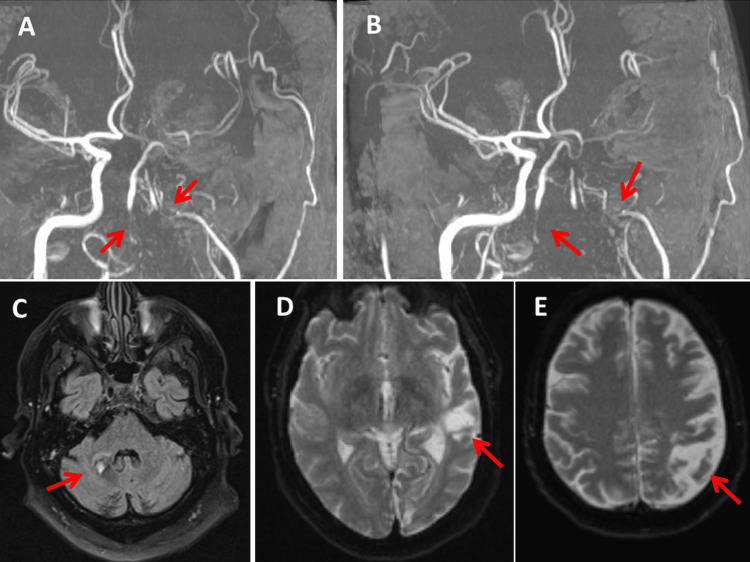
MRI of the index patient’s brain at the age of 54, with stenosis of the basilar artery and occlusion of the distal internal carotid artery (A and B) (red arrows) and old ischemic lesions in the left posterior cerebral artery and the territories of the left middle cerebral artery (D and E) (red arrows). MRI: magnetic resonance imaging

**Figure 3:**
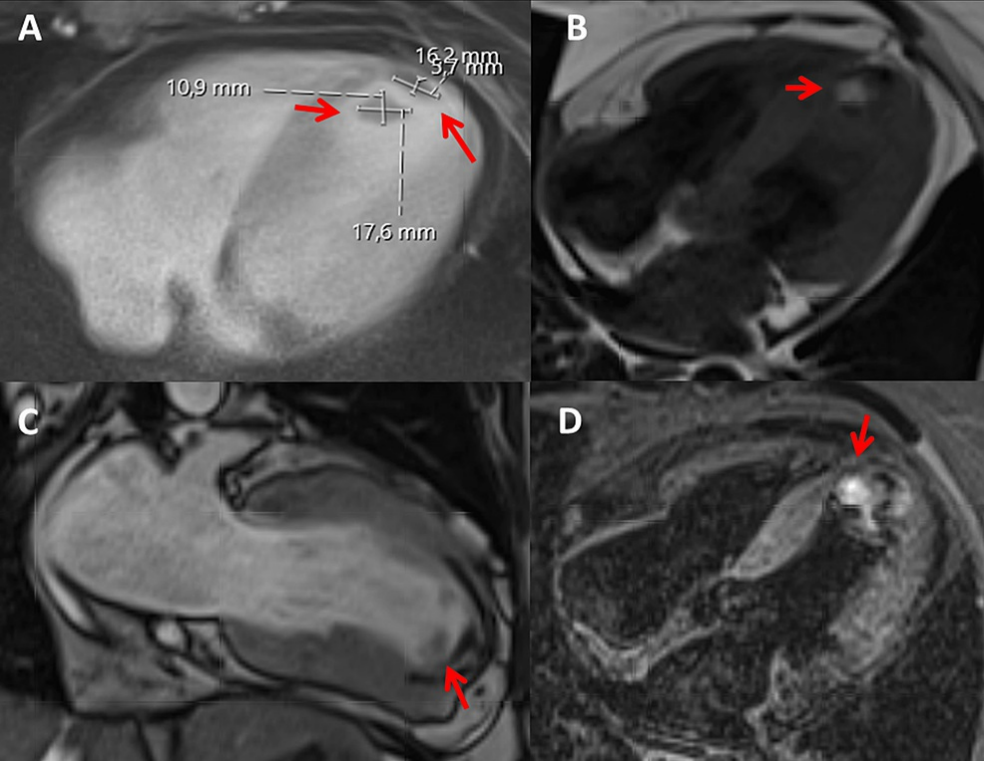
Cardiac MRI of the index patient’s heart at age 54 showing myocardial thickening and two organized apical thrombi at different modalities and projections (A-D) (red arrows). MRI: magnetic resonance imaging

At the age of 54, under phenprocoumon, he was admitted in a state of neglect (the patient had been homeless repeatedly since 1999) with dysarthria for six days prior to admission. He had no fever and no tachycardia or tachypnea, and his blood pressure was within normal limits. He had bilateral leg edema, stasis dermatitis, bilateral leg ulcers with maggot infestation of the left leg, and sacral decubitus ulcer. Electrocardiogram (ECG) showed sinus rhythm. Blood tests revealed elevated C-reactive protein (CRP), elevated lactate dehydrogenase (LDH), hypokalemia, hypocalcemia, hyperparathyroidism, hypothyroidism, mildly elevated transaminases, hyperlipidemia, and protein deficiency. The INR value at admission was 1.3. Other coagulation parameters were within the normal range. Cerebral computed tomography (CT) revealed a subacute ischemic stroke with secondary, non-mass bleeding in the right cerebellar hemisphere and multiple old ischemic lesions in other territories (Figure 1). Cerebral magnetic resonance imaging (MRI) revealed multiple old ischemic lesions in the territories of the left superior cerebellar artery, left posterior cerebral artery, and left middle cerebral artery territory (Figure 2). Magnetic resonance angiography showed left distal internal carotid artery occlusion and basilar artery stenosis (Figure 2). Carotid ultrasound showed filiform stenosis of the external carotid arteries but normal flow in the proximal internal carotid arteries. Transthoracic echocardiography revealed ischemic cardiomyopathy with apical hypokinesia and postischemic scarring in the left anterior descending artery territory with preserved systolic function. Cardiac MRI showed organized apical thrombi (Figure 3). Cardiologists did not indicate coronary angiography. Phenprocoumon was initially discontinued and replaced with low-dose heparin (60 mg/d). After seven weeks in the hospital, he developed diplopia. Neurological examination revealed dysarthria, sub-innervation of the left corner of the mouth, coated tongue, cervical tenderness, generally reduced tendon reflexes, left upper extremity ataxia, pointing past right, marked trophic dysfunction in both lower legs, blurred vision, nausea, and orthostasis after sitting up and inability to stand. Dorsalis pedis pulses were not palpable. After complete resorption of the bleeding, heparin was replaced with dabigatran (300 mg/d). Additionally, L-thyroxin, quetiapine, furosemide, simvastatin, and pantoprazole were prescribed.

## Discussion

The index patient is of interest for the combination of hereditary protein S deficiency and APC resistance, which most likely contributed to the development of recurrent thrombosis and recurrent subclinical ischemic stroke. Protein S deficiency was thought to be hereditary because it was detected at the first DVT at age 36 and because of his family history of the disorder. Whether the Leiden mutation was inherited or sporadic remains speculative, as other family members have not previously been available for genetic testing. However, the homozygosity of the variant is more indicative of inherited APC resistance. The multiple ischemic strokes were attributed to a combination of recurrent embolism due to coagulopathy and atherothrombosis due to the patient’s multiple cardiovascular risk factors. Arguments for an embolism are the two hereditary coagulopathies and the presence of intracardiac thrombi. However, the apical thrombi were scored as organized and nonvolatile. Acquired thrombophilic disorders such as antiphospholipid antibody (APLA) syndrome, paroxysmal nocturnal hemoglobinuria, myeloproliferative disorders, and elevated blood coagulation factors have been ruled out as the cause of coagulopathy [6]. In favor of atherosclerosis is the presence of classical cardiovascular risk factors, such as arterial hypertension, smoking, hyperlipidemia, and the presence of generalized atherosclerosis affecting the intracranial, extracranial, coronary, and leg arteries. In any event, however, blood pressure was well controlled during the two months since admission without the need for antihypertensive medication, and he had been a nonsmoker since admission. Hyperlipidemia (low-density lipoprotein (LDL): 173 mg/dL) normalized with diet. He lost 40 kg within the first two months of his hospital stay. There was no evidence of dehydration or renal insufficiency on admission or during the hospital stay. Apart from the TIA at age 14, his strokes were subclinical as he had a negative history of ischemic stroke.

Diplopia has been related to basilar artery stenosis. The polyneuropathy was attributed to previous chronic alcoholism because other causes were considered unlikely after the most common causes of polyneuropathy, such as diabetes, renal insufficiency, malignancy, intoxication, vitamin deficiency, medication, and immunological disorder, were ruled out. The hypertrophy of the left ventricular myocardium was attributed to previous arterial hypertension rather than hypertrophic cardiomyopathy since the patient had indications for ischemic heart disease. The presence of intraventricular thrombi was attributed to the two coagulation disorders rather than heart failure or endocarditis since the latter did not occur during the hospital stay. Ventricular thrombi have been described in particular in connection with protein S deficiency [7-9]. APC resistance does not appear to be a risk factor for the development of ventricular thrombi [10]. However, it cannot be ruled out that the old ventricular thrombi originated from a previous, subclinical myocardial infarction. Therefore, it is recommended to undergo coronary angiography in patients with ventricular thrombi and apical hypokinesia.

In view of the many acquired atherothrombotic risk factors in addition to hereditary hypercoagulability (lifestyle, obesity, immobility, atherosclerosis, and lack of sufficient secondary prevention (aspirin in addition to vitamin K antagonist (VKA) and statin)), noncompliance to VKA therapy, and lack of intervention for minimizing the risk of recurrence (carotid endarterectomy, coronary angioplasty, and cava filter), it cannot be ruled out that they have contributed to the many thrombotic and ischemic events. Obesity, in particular, has been identified as a risk factor for DVT and pulmonary embolism [11]. However, TIA, DVT, and pulmonary embolism occurred when the patient was not obese (ages 14, 38, 42, and 48). He became obese with increasing immobility for the last two years prior to admission at age 54. He has not experienced DVT since becoming obese. The patient was able to walk with a walker until he suffered the cerebellar stroke, leading to dizziness and an inability to stand. When switching from vitamin K antagonist (VKA) to dabigatran, it must be noted that a recent consensus statement from the European Society of Cardiology recommends that in patients with a BMI of >40 kg/m^2^, the effect of dabigatran should be monitored by measuring the diluted thrombin time, and if not increased, dabigatran should be replaced by VKA [12].

## Conclusions

This case shows that the simultaneous presence of two hereditary coagulation disorders, such as protein S deficiency and APC resistance, can lead to multiple venous thromboses and multilocular ischemic strokes. Risk factors for atherosclerosis may have an over-additive impact on stroke risk. An appealing therapeutic option for patients with low compliance and indication for long-term anticoagulation due to two hereditary clotting disorders is the replacement of long-term anticoagulation with a VKA with a direct oral anticoagulant.
